# Spatial Distribution of Dominant Arboreal Ants in a Malagasy Coastal Rainforest: Gaps and Presence of an Invasive Species

**DOI:** 10.1371/journal.pone.0009319

**Published:** 2010-02-19

**Authors:** Alain Dejean, Brian L. Fisher, Bruno Corbara, Raymond Rarevohitra, Richard Randrianaivo, Balsama Rajemison, Maurice Leponce

**Affiliations:** 1 Écologie des Forêts de Guyane, Centre National de la Recherche Scientifique, Unité Mixte de Recherche 8172, Campus agronomique, BP 709, Kourou, France; 2 Department of Entomology, California Academy of Sciences, San Francisco, California, United States of America; 3 Laboratoire Microorganismes Génome et Environnement, Centre National de la Recherche Scientifique, Unité Mixte de Recherche 6023, Université Blaise Pascal, Aubière, France; 4 Département de Recherches Forestières et Piscicoles, BP 904, Antananarivo, Madagascar; 5 Parc Botanique et Zoologique de Tsimbazaza, BP 4096, Antananarivo, Madagascar; 6 Biological Evaluation Section, Royal Belgian Institute of Natural Sciences, Brussels, Belgium; Field Museum of Natural History, United States of America

## Abstract

We conducted a survey along three belt transects located at increasing distances from the coast to determine whether a non-random arboreal ant assemblage, such as an ant mosaic, exists in the rainforest on the Masoala Peninsula, Madagascar. In most tropical rainforests, very populous colonies of territorially dominant arboreal ant species defend absolute territories distributed in a mosaic pattern. Among the 29 ant species recorded, only nine had colonies large enough to be considered potentially territorially dominant; the remaining species had smaller colonies and were considered non-dominant. Nevertheless, the null-model analyses used to examine the spatial structure of their assemblages did not reveal the existence of an ant mosaic. Inland, up to 44% of the trees were devoid of dominant arboreal ants, something not reported in other studies. While two *Crematogaster* species were not associated with one another, *Brachymyrmex cordemoyi* was positively associated with *Technomyrmex albipes*, which is considered an invasive species—a non-indigenous species that has an adverse ecological effect on the habitats it invades. The latter two species and *Crematogaster ranavalonae* were mutually exclusive. On the other hand, all of the trees in the coastal transect and at least 4 km of coast were occupied by *T. albipes*, and were interconnected by columns of workers. *Technomyrmex albipes* workers collected from different trees did not attack each other during confrontation tests, indicating that this species has formed a supercolony along the coast. Yet interspecific aggressiveness did occur between *T. albipes* and *Crematogaster ranavalonae*, a native species which is likely territorially dominant based on our intraspecific confrontation tests. These results suggest that the Masoala rainforest is threatened by a potential invasion by *T. albipes*, and that the penetration of this species further inland might be facilitated by the low density of native, territorially dominant arboreal ants normally able to limit its progression.

## Introduction

Ants dominate the animal communities of tropical rainforest canopies in terms of biomass and number of individuals and have become adapted to the climatic conditions of this environment. Their ecological success is made possible by the fact that most arboreal ants are at least partially herbivorous – feeding on extra-floral nectaries, food bodies, pollen, epiphylls, and sap – and they are also “cryptic herbivores” that feed on hemipteran honeydew [1]–[3].

In these canopies, a few “territorially-dominant” arboreal ant species are characterized by very populous colonies of up to several million workers, large and/or polydomous nests, and an absolute intra- and interspecific territoriality. The territories of these species are distributed in a mosaic pattern, creating what have become known as “arboreal ant mosaics” [4]–[8]. Territorially-dominant arboreal ants tolerate within their territories the presence of “non-dominant” species with small colonies of hundreds of workers. Under favorable conditions, some of these non-dominant species are able to develop larger colonies that behave like territorially-dominant ants. Such colonies are known as “sub-dominant”. Two colonies of territorially-dominant arboreal species have been known to share the same territory; these “co-dominant” species generally have complementary rhythms of activity: one is often diurnal, while the other is nocturnal [4], [7].

Types of arboreal ant nesting sites include: pre-existing cavities (typically branches bored out by xylophagous insects and used by opportunistic species); galleries bored by carpenter ants; silk nests built by weaver ants; and carton nests [7]–[9]. Certain myrmecophytes (plants sheltering ant colonies in hollow structures such as leaf pouches, hollow branches and thorns [9]) grow large enough to reach the canopy, enabling their associated plant-ants to become a part of the ant mosaic [7], [8]. Also, along with territorially-dominant species, invasive ants may form a new component in some ant mosaics, as *Technomyrmex albipes* (Dolichoderinae) does in Southeast Asia [10].

Of the approximately 14,000 ant species known, about 150 “tramp species” have been transported and introduced into many parts of the world through human activity, but only some of them have become invasive [11]. The aptitude for invasiveness primarily stems from an intrinsic ability to shift their colony structure. Invasive ants that form a multi-colonial social structure in their native range can switch to supercoloniality (the formation of colonies extending over extremely large areas) in their introduced, and, among certain species, also in their native range. Unicoloniality refers to a population forming a single supercolony over several hundred or even thousand kilometers [11]–[13]. All of these huge colonies are based on extreme polydomy (multiple nests) and polygyny (multiple queens), so that the workers' relatedness is very low in most supercolonies [12], [13]. Ants are often highly territorial because they can recognize kin through differences or similarities in their cuticular hydrocarbons. Those living within each supercolony are tolerant of one another, freely mixing between different nests even if they are tens of kilometers apart [11]–[15]. The most plausible hypothesis explaining the absence of aggressiveness between workers from the same supercolony is that recognition and aggressiveness may be genetically based. For this reason, species that form supercolonies are believed to share similar or identical heritable recognition cues that surpass the influence of the environment on the composition of their cuticular hydrocarbons [16]–[18].

Together, the absence of natural enemies for introduced species (enemy release hypothesis) and a reduction in the costs associated with intraspecific territoriality for supercolonies permit more energy to be allocated to the production of workers [11]. This results in high worker densities that can monopolize habitat by excluding other ant species through exploitative and interference competition [11], [19], [20].

Consequently, invasive ants are among the most harmful bioinvaders known. They penetrate ecosystems by disassembling the native ant community, and occasionally even eliminate other species. By lowering native ant abundance and diversity, they directly or indirectly affect all other organisms that depend on those species, and modify large geographical regions by disrupting native communities [11], [21]. Among the potential invasive ant species reported in Madagascar is the dolichoderine ant *Technomyrmex albipes* which is particularly abundant along the coast [22]. Likely native to the Pacific Islands, *T. albipes* is an extremely successful tramp species that nests both terrestrially and arboreally with workers that attend a wide range of hemipterans [22]. Its success is also facilitated by the mode of reproduction it shares with other species from the *albipes* group: the reproductive castes include ergatoid females and males in addition to alates of both sexes, which facilitates the formation of supercolonies [22]. In the past, other species from the *albipes* group were often misidentified as *T. albipes* (see p. 70 in Bolton [22]); for example, the ants identified in references made to *T. albipes* in Terayama [23] and Tsuji & Yamauchi [24] are, in fact, *T. brunneus* [22].

Initially, the aim of this study was to verify during a snapshot field survey whether or not an ant mosaic existed in a Malagasy rainforest. To do this, we had planned to study three transects, one along the coast, and two others inland. Because we observed *T. albipes* workers on numerous trees over several kilometers along the coast, we decided to investigate the possible presence of a supercolony. We thus verified whether all of the trees were occupied by this species, and if columns of workers interconnected these trees over large distances along the coast. We conducted standard behavioral assays to establish whether workers gathered from distant areas showed aggression toward one another. We also tested *T. albipes* worker aggression against a native arboreal species thought to be territorially dominant.

## Results

### Tree and Ant Species Composition along the Three Transects

Twelve tree species (seven families) were found along the “coastal transect” (situated along the shore), but three species represented 81.3% of the cases (N = 150): *Barringtonia butonica* (Lecythidaceae), *Bruguiera gymnorhiza*, and *B. sexangula* (Rhizophoraceae) (N = 65, 36 and 21 trees, respectively). In addition, 64 clusters of *Medinilla* sp., an epiphytic Melastomataceae, were noted on 46 trees (30.7%; N = 150). Large numbers of *T. albipes* workers patrolled the branches, foliage, and trunks of all of the trees, and columns of workers following trails traversed the ground between trees whose crowns were not interconnected. We also noted the presence of several ant species with small colonies (with the exception of introduced *Brachymyrmex cordemoyi*) that typically nested under *Medinilla* clusters; these colonies were tolerated by *T. albipes* (Data set S1).

**Figure 1:**
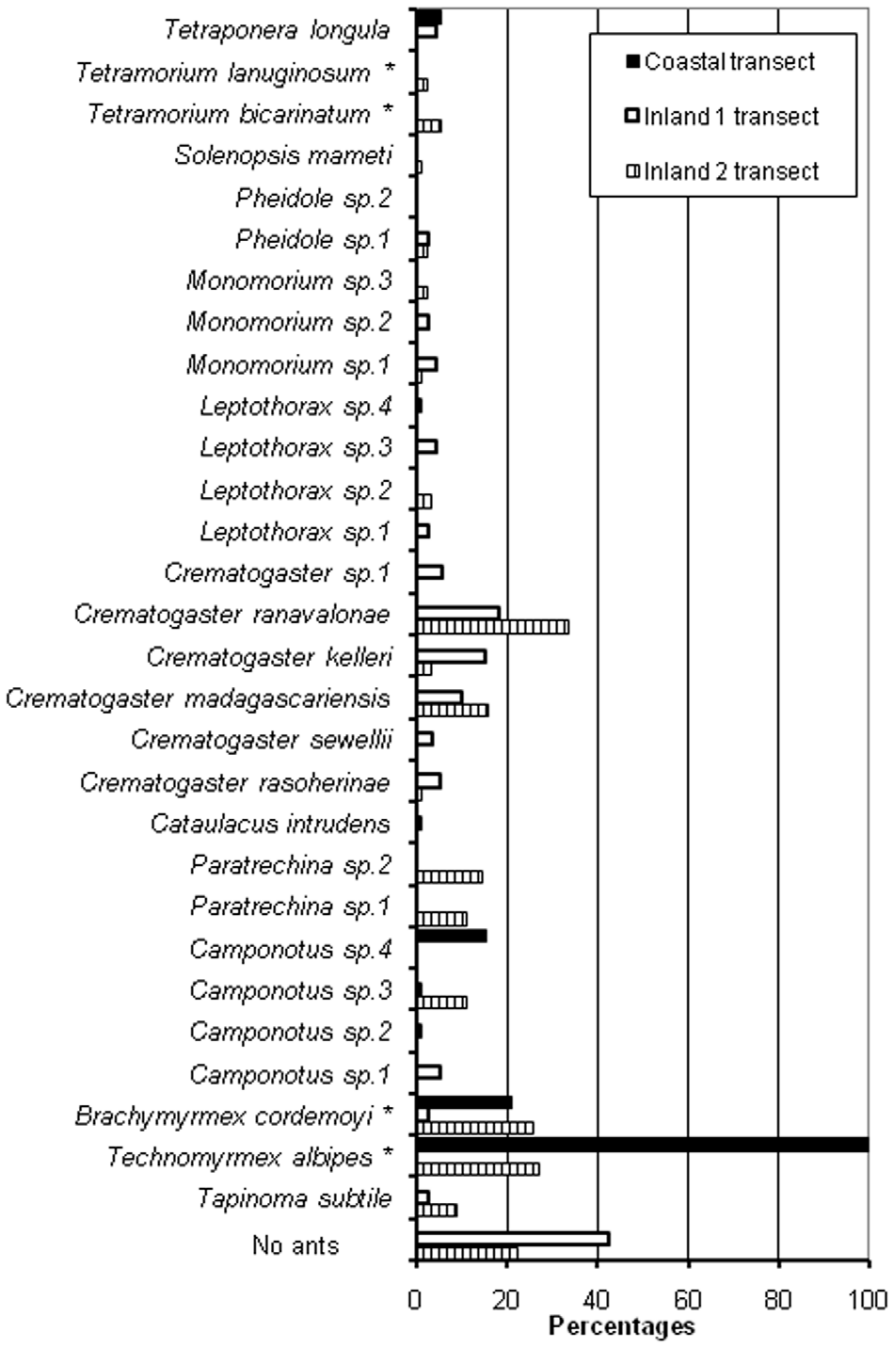
Relative frequency for the 29 ant species recorded in the coastal transect and the two inland transects. Because one tree can shelter several ant species, the total percentages per transect can surpass 100%.* introduced species.

In the two “inland transects”, where we recorded 73 tree and eight liana species, the above-cited three tree species were absent, while *Medinilla* sp. was noted only once. There was little similarity in tree species between these two inland transects (Chao-Jaccard abundance-based index: 0.20±0.27 [mean ± SE]; Fig. 1). Among the 29 total ant species gathered, 18 species were found in both inland transects, and nine were shared species (Chao-Jaccard incidence-based similarity index = 0.58±0.18; Fig. 1). *Crematogaster ranavalonae* was the most common species in both inland transects (Fig. 1) and was found on 35 tree species belonging to 20 different families (Data set S1). Yet, the area studied was characterized by the absence of arboreal ants in the crown of 44% (54/120) and 26% (23/89) of the trees in the inland 1 and inland 2 transects, respectively (Data set S1; Fig. S1), but the difference is not significant (Fisher's exact-test: P = 0. 17).

By climbing trees or using the canopy sledge (a device carried by a blimp), we noted that the colonies of only nine ant species were large enough to occupy at least one tree crown; they occupied adjacent trees in certain cases, as we observed large numbers of workers passing along the branches from one tree to another (see Fig.S1). We verified this by cutting off relatively large branches, which permitted us to sample workers. Here we also noted the presence of other ant species with much smaller colonies (hundreds of workers) corresponding to the status of non-dominant species (see Data set S1). Some were represented by a few foraging workers, while in other cases we found entire colonies represented by at most several hundred individuals after we cut the branches into smaller pieces with a machete and pruning scissors.

Of the nine ant species with large colonies, only *B. cordemoyi* and *T. albipes* (two introduced, opportunistic nesters that frequently share trees and even nest areas) were recorded along the coast. In the inland transects, five native arboreal species had colonies large enough to possibly be territorially dominant. *Crematogaster rasoherinae*, *Cr. madagascariensis* and *Cr. kelleri* nested in hollow branches several centimeters wide. Very populous colonies of *Crematogaster* sp.1 nested in the naturally hollow branches of *Vitex beraviensis* (Lamiaceae; trees A7 and A12; Data set S1 and Fig. S1), while numerous foraging workers invaded lianas and adjacent trees, demonstrating that their territory was not limited to their host plant. The relatively large *Cr. ranavalonae* workers built ovoid carton nests 35 to 60 cm in height and 25 to 50 cm in diameter. These nests, reminiscent of those belonging to territorially-dominant arboreal African *Crematogaster*, were distributed over the main branches of several adjacent trees and interconnected by trails (polydomous colonies). Colonies of the last two species, *Tapinoma subtile* and *Camponotus* sp.1, occupied the entire crown of a tree on occasion, but were most frequently associated with other ant species (co-dominance).

### Intra- and Interspecific Aggressiveness between *Technomyrmex albipes* and *Crematogaster ranavalonae* Colonies

While searching for evidence of the presence of *T. albipes* along 4 km of coast, we noticed that columns of workers interconnected all trees through their foliage or along trails on the ground.

When confronted with *Cr. ranavalonae* individuals, *T. albipes* workers initiated 40% of the combats (N = 60) and fought and bit, which corresponds to strongly agonistic behavior. By contrast, *T. albipes* workers showed a complete lack of intraspecific aggressiveness (Table 1), even when individuals were gathered from areas separated by up to 8.4 km. This was not the case between *Cr. ranavalonae* workers belonging to different areas, and which were frequently involved in reciprocal full attacks.

**Table 1:** Values for aggressiveness during one-on-one confrontations between workers.

I	Sites	**A**	**B**	**C**	**D**	**E**	**F**
	**F**, **H** for *Cr. ranavalonae*	2.9	2.7	2.9	3.7	3.0	3.1
II	Sites	**A**	**B**	**C**	**D**	**E**	**F**
	**A**	1.1	1.1	1.2	1.1	1.0	1.1
	**B**		1.2	1.4	1.2	1.2	1.1
	**C**			1.2	1.3	1.4	1.2
	**D**				1.3	1.1	1.0
	**E**					1.1	1.1
	**F**						1.1
III	Sites	**F**	**G**	**H**			
	**F**	1.1	3.7	3.3			
	**G**		1.0	3.2			
	**H**			1.0			

(I) Interspecific confrontations between *Technomyrmex albipes* (colonies from areas A–F) and *Cr. ranavalonae* workers (from areas F, G). Areas A–E correspond to a total of ca. 8.4 km of shoreline, while area F is situated 1.7 km inland. Aggressiveness values were recorded only for *T. albipes* workers and calculated for cases where they initiated the encounters. Statistical comparisons (Kruskal–Wallis tests); *T. albipes vs. Cr. ranavalonae*: H^5^_60_ = 9.5; P = 0.092. (II) Intraspecific aggressiveness between *T. albipes* workers; control lot or individuals from the same tree: H^5^_60_ = 3.4; P = 0.80; experimental lot or individuals from two different areas: H^14^_150_ = 13.2; P = 0.51. Comparison taking into account inter- and intraspecific confrontations: H^2^_150_ = 1573.1; P<0.0001; Dunn's multiple comparison tests, *T. albipes* workers confronted with *Cr. ranavalonae vs. T. albipes* workers from the control or the experimental group: P<0.001 in both cases; confrontations between *T. albipes* workers (control *vs.* experimental lot): P>0.05. (III) Intraspecific aggressiveness between *Cr. ranavalonae* workers gathered from three different areas (F, G, H). Workers from the same area/colony were not aggressive with each other, while this was not the case for those from different areas. Statistical comparisons (Kruskal–Wallis tests): H^5^_60_ = 49.46; P<0.0001; Dunn's multiple comparison tests, workers from the same area: P>0.05 in all cases; from two different areas: P<0.05; workers from same area *vs.* from two different areas (control *vs.* experimental lots): P<0.01.

To study whether group size influences aggressiveness, we also transported groups of ca. 200 foraging *T. albipes* workers and installed them among conspecifics in a different zone of the same tree (control) or on a tree situated several hundreds meters away (experiment). The results confirmed those recorded during individual bouts, as the transferred individuals were integrated into nearby worker columns in all cases. The same experiments conducted with *Cr. ranavalonae* workers resulted in the absence of aggressiveness between workers originating from different parts of the same tree crown, but all of those transferred to distant trees were spread-eagled and then killed by resident ants, illustrating the existence of intercolonial aggressiveness.

### Looking for an Ant Mosaic Inland

For the nine species with large colonies, which were also the most frequently encountered, the null model analysis indicated that species co-occurrences were more frequent than might be expected in the inland transects (P<0.05 and P = 0.07, respectively) and in the coastal transect (P<0.001) due to chance. More specifically, we found a significant positive association between *Cr. madagascariensis* and *Cr. kelleri* in the inland 1 transect. In the inland 2 transect, we found a significant positive association between *T. albipes* and *B. cordemoyi*, but a negative association between *Cr. ranavalonae* and *T. albipes* and between *Cr. ranavalonae* and *B. cordemoyi* (Table 2). In the coastal transect, we found a significant positive association between *B. cordemoyi* and *Camponotus* sp.4. Note that numerically-dominant native species present in both inland transects were absent here.

**Table 2:** Associations between the most frequent species (relative frequency >5%) from the three transects sorted by decreasing rank of occurrence and tested using *Chi*-square tests (1 d.f., Yates' correction).

		Relative frequency	Species	1	2	3	4	5
Coastal	1	70%	*Technomyrmex albipes*					
transect	2	15%	*Brachymyrmex cordemoyi*	X				
	3	9%	*Tetraponera longula*	X	0			
	4	6%	*Camponotus* sp.4	X	+	0		
Inland 1	1	21%	*Crematogaster ranavalonae*					
transect	2	19%	*Crematogaster kelleri*	0				
	3	12%	*Cremato. madagascariensis*	0	+			
	4	6%	*Crematogaster rasoherinae*	0	0	0		
	5	6%	*Camponotus* sp.1	0	0	0	0	
	6	5%	*Crematogaster* sp.1	0	0	0	0	0
Inland 2	1	20%	*Crematogaster ranavalonae*					
transect	2	16%	*Technomyrmex albipes*	[−]				
	3	15%	*Brachymyrmex cordemoyi*	[−]	+			
	4	10%	*Cremato. madagascariensis*	0	0	0		
	5	5%	*Tapinoma subtile*	0	0	0	0	

Symbols indicate the nature of the association.: +: positive, [−] negative, 0: not significant. X indicates that the test is meaningless since *Technomyrmex albipes* is present in every sample.

## Discussion

In addition to the lack of intra-specific aggressiveness between workers gathered from places separated from each other by up to 8.4 km, the presence of trails between trees along the shore supports our argument that a *T. albipes* supercolony exists in Madagascar [see 25] and probably covers an area larger than we were able to explore. Genetic data would be necessary for definitive proof of a supercolony's existence [13], [17], [26].

As reported for *T. brunneus* [23], environmental conditions along the coast may favor the formation of a *T. albipes* supercolony (see also the 12 out of 14 Malagasy locales cited by Bolton [22]). The present study also suggests that *T. albipes* can spread inland through patches where resident workers will not fight with those from the shore. The extension of these patches can lead to interconnections and the formation of a huge colony. Note that the populations in supercolonies do not necessarily span a contiguous area [27], so that *T. albipes* from the inland transects could belong to the same supercolony as those from the coast, and these entities might even be interconnected from time to time. Indeed, *T. albipes*, described as invasive at least once [28], has been reported inland in Madagascar [22], Borneo and Malaysia [10].

Tests of species co-occurrences revealed no ant mosaic structure since dominant species were generally independently distributed or positively associated. Nevertheless, our survey yielded several arboreal ant species likely to be territorially dominant, in particular the carton-building *Cr. ranavalonae*. This species has polydomous colonies spread over several trees and shows strong intraspecific and interspecific aggressiveness towards *T. albipes*. Its habits are reminiscent of the carton-building *Crematogaster* species that participate in African rainforest ant mosaics [7], [8]; the smaller ovoid nests are likely adapted to the harsh climatic conditions of the Masoala Peninsula. *Crematogaster rasoherinae* and *Cr. kelleri* workers, which colonize several adjacent trees of different species, are likely to bore galleries into branches like the African Myrmicinae *Atopomyrmex mocquerisii* [29]. That *Crematogaster* sp.1 nests in the naturally hollow *V. beraviensis* branches indicates that this tree species could be a myrmecophyte like several others of the genus *Vitex* [30]. We also recorded a nocturnal species, *Camponotus* sp.1, which shared trees with different dominant species (co-dominance). Finally, *B. cordemoyi*, which - like other species of the genus - has very tiny workers, was associated with *T. albipes*.

Both inland transects were characterized by a high proportion of trees devoid of dominant arboreal ants (areas where no arboreal ants were recorded, but discrete species might have been present), something never before reported to the best of our knowledge. Indeed, all other studies conducted so far in the humid tropics have found that most, if not all, canopy trees sheltered ants, regardless of whether or not these ants were dominant. The presence of numerous trees devoid of native dominant arboreal ants cannot be ascribed to an ability to repel arboreal ants, as all of these tree species sheltered ant colonies elsewhere in the transects (see Data set S1). Rather, several non-exclusive factors might be involved, such as canopy structure, the size of the colonies' territories, and climatic impacts such as particularly strong storms that destroy exposed arboreal nests on this peninsula. The latter may explain why we noted only one species, *Cr. ranavalonae*, with external nests; all other species nest in hollow branches, the cavities formed by rough bark or the root area of the epiphyte *Medinilla*.

Thanks to their number and aggressiveness, *T. albipes* workers might have excluded native dominant arboreal ants from the coast (present inland, they were absent from the coast). An invasive process likely had begun, as numerical dominance often favors invasive species through the rapid recruitment of relatively aggressive nestmates that eliminate native species through exploitative and interference competition [11], [19], [20]. The fact that numerous inland trees were apparently devoid of ants could favor the explanation that invasive species had penetrated the area (see [11] about unsaturated island ecosystems). Indeed, *T. albipes* was noted in both inland transect 1 and ca. 2 km inland. The success of invasive ants is associated with the absence of intraspecific aggressiveness due to the formation of supercolonies accompanied by high interspecific aggressiveness [11]. This is true for *T. albipes*, as (1) workers gathered inland did not fight with those from the shoreline; and (2) all were aggressive toward *Cr. ranavalonae* individuals. In Southeast Asia, where territorially-dominant species such as *Oecophylla smaragdina* are present, *T. albipes* colonies are involved in the formation of an ant mosaic [10]. In this case, although *T. albipes* successfully penetrated inland, its invasive action seems limited by native, territorially-dominant arboreal ants.

Beachhead invasions by ants have already occurred on the Galapagos, Hawaii, Mauritius, New Caledonia and Christmas Island, and pose a significant conservation concern [11], [31], [32]. Although we noted the presence of arboreal ant species likely to be territorially dominant, the distribution of their territories was very loose, a factor that could favor the penetration inland of *T. albipes*. The forest canopy of the Masoala National Park could be under threat from *T. albipes*, which has already established itself along the coast and could easily spread inland.

## Materials and Methods

### Ethics Statement

This work was conducted according to relevant national and international guidelines.

### The Transects

For this study, conducted between 12 October and 10 November 2001 in a rainforest on the western coast of the Masoala National Park around the estuary of the Tampolo River (15° 43′ 45″S, 49° 57′ 38″E), Madagascar, we surveyed three belt transects. For all of the surveys, we cut off two to four relatively large sections (diameter>10 cm) of branches from each tree. Because arboreal ants mark these branches as part of their territories, they remained on the branches for more than one hour after these sections were removed. Any ants found on or under tree bark or in hollow twigs were collected with aspirators. The trees were typically identified based on the flowers and/or fruit attached to the branches. We used the single rope technique to reach the canopy in the first two transects (see [33]) and the canopy sledge in the third transect. The canopy sledge (“*Luge des cimes*”) is an inflatable device carried by a blimp that can transport two persons from treetop to treetop [34]. Branches harvested via the single rope technique were collected between 8:00 and 12:00, and at night (between 21:30 and 23:30) on trees apparently devoid of ants during the day. Branches harvested via the canopy sledge were collected between 5:30–7:30 in low wind conditions.

Our aim was to rapidly assess the distribution of the dominant arboreal ants over a wide area, not to conduct an exhaustive inventory of the arboreal ant assemblage. Preliminary tests showed that clipping two to four large branches from each tree was sufficient to capture opportunistic nesting, diurnal, dominant arboreal ants (the nests made by weaver or carton-building ants are easily detectable). A complete survey of all of the ant colonies living in each tree would, however, require a much greater effort incompatible with the conditions of a snapshot study.

The first, or “coastal,” transect (20 m wide at ground level; 175 m long; altitude 1–3 m; all tree crowns inspected), included 150 trees, 6–20 m tall, and was situated along the shore, beginning on the right bank of the estuary of the Tampolo River. The second, or “inland 1” transect (20 m wide; 175 m long and with an additional area, Fig. S1; altitude 15 m), included 120 trees about 30 m tall and was parallel to the coast 100 m away, beginning 400 m from the right bank of the estuary of the Tampolo River. The third, or “inland 2” transect, included 89 adjacent trees about 30 m tall and was located 2 km inland (10–15 m wide; ca.100 m long; altitude 35 m).

All plants were morphotyped at least to family save for five dead or unrecognizable trees in the inland 1 transect. Ants were preserved in 70% ethanol for later identification to species or morphospecies. Voucher specimens were deposited at the Bibikely Biodiversity Center, Parc Botanique et Zoologique de Tsimbazaza, BP 4096, Antananarivo, Madagascar, and the Department of Entomology, California Academy of Sciences, San Francisco, USA.

### Intra- and Interspecific Aggressiveness between *Technomyrmex albipes* and *Crematogaster ranavalonae* Colonies

After surveying the coastal transect, we checked for the presence of a *T. albipes* supercolony along 4 km of coast. We observed whether or not the columns of *T. albipes* workers went from one tree to another (ca. 3400 trees). In addition, we employed the standard behavioral assay created by Suarez *et al.* [26, see also 16, 25] to test the aggressiveness between *T. albipes* individuals collected from different sites. We paired workers originating from six sites (one tree per site) or from the same tree at each site (control). Five of these sites (sites A–E) were situated along 8.4 km of coast. Three sites (A–C) were located to the north of the Tampolo River estuary between Andronabé and ca. 0.7 km to the south of Camp Tampolo; two others (D and E) were situated to the south of the estuary (see map in *Radeau des cimes* 2000 [35]). The distance between sites A and B was ca. 6.3 km and the other sites were spaced at intervals of 0.7 km. The sixth site (F) was situated 1.7 km inland across from the fifth site.

For the tests, two individual workers were placed in a neutral arena (Ø: 6 cm; height: 7 cm) whose walls were coated with fluon® to prevent the ants from climbing out. We scored interactions between the workers over a 5 minute period on a scale from 1 to 4: 1 = physical contact, but no aggressive response (may include antennation or trophallaxis), 2 = avoidance (the ants touch, and one or both recoils and runs in the opposite direction), 3 = aggressiveness (biting legs or antennae), and 4 = fighting (prolonged biting, pulling, or gaster bending by one or both ants). We repeated the confrontations 10 times, retaining the highest value noted each time, and used each worker only once.

For comparison, the behavior of *T. albipes* from each site was recorded during confrontations with *Cr. ranavalonae*, the most frequent species collected in the inland transects. We also set up intraspecific confrontations between *Cr. ranavalonae* workers belonging to three colonies (sites F, G, H) separated by more than 1 km. Levels of aggressiveness between sample pairs were compared using the Kruskal-Wallis test (GraphPad Prism 4.0 Software).

Because there was a demonstrated effect of group size on aggressiveness [25], [36], in a complementary experiment, we transported foraging workers from two zones of the same tree from sites C, E and F (control), and between trees situated on the three sites; 10 groups of approximately 200 workers were transported in each case. The same experiment was conducted with *Cr. ranavalonae* workers from sites F, G and H.

### Comparing the Two Inland Transects and Testing the Existence of an Ant Mosaic

The similarity between samples was calculated using the Chao-Jaccard abundance-based similarity index for trees, and the Chao-Jaccard incidence-based similarity index for ants. These indices are appropriate for the comparison of incompletely sampled species-rich communities [37]. Standard errors for the Chao-Jaccard estimators were computed through 200 bootstrap procedures using EstimateS 7.5 software.

Global trends in species associations were investigated using a fixed-equiprobable null model and the C-score co-occurrence index available in the EcoSim software [38]. The fixed-equiprobable algorithm, appropriate for data-matrices with unoccupied sites, maintains the species occurrence frequencies and considers all sites (trees) equiprobable [39]. Tests not shown here confirmed that the outcome of the null model analysis was not altered by including trees not occupied by ants. The C-score index used in combination with the fixed-equiprobable algorithm has generally good statistical properties and is not prone to false positives [39].

Specific associations between the most common species were tested using *Chi*-square tests (Yates' correction). During sampling, we noted when ant colonies were gathered from the same tree crown, when host trees were adjacent, and whether an ant species was represented by a small or a large colony. When the colonies of two dominant species occupied different areas of the same tree crown, two different territories were distinguished (i.e., some of the large branches sampled were occupied by one dominant species, and others by a second dominant species), and the species were not considered co-occurring for the species association analysis [6], [7].

## Supporting Information

Figure S1 Schematic representation of the distribution of ant species recorded on trees from the inland 1 transect.
(0.07 MB DOC)

Data set S1 Series and tree species monitored in the inland 1 transect (A1-A120) and the inland 2 transect (L1-L89), and ant species recorded nesting in tree crowns. // : cases when ant species were recorded on different branches of the same tree (two different territories on the same tree). For the inland transects we provide the trees' code in the first column (see also Figure S1 for inland transect 1); for the coastal transect we provide only the number of trees.
(0.24 MB DOC)
