# Supplementary material for: Spatial Distribution of Dominant Arboreal Ants in a Malagasy Coastal Rainforest: Gaps and Presence of an Invasive Species
Source: PLoS One. 2010 Feb 19;5(2):e9319. doi: 10.1371/journal.pone.0009319 (PMC2824834; doi:10.1371/journal.pone.0009319)
Supplement: Data set S1 — Series and tree species monitored in the inland 1 transect (A1-A120) and the inland 2 transect (L1-L89), and ant species recorded nesting in tree crowns. // : cases when ant species were recorded on different branches of the same tree (two different territories on the same tree). For the inland transects we provide the trees' code in the first column (see also Figure S1 for inland transect 1); for the coastal transect we provide only the number of trees. (0.24 MB DOC) [file pone.0009319.s002.doc]

**Data set S1. Series and tree species monitored in the** **inland 1 transect (A1-A120) and the inland 2 transect (L1-L89), and ant species recorded nesting in tree crowns**. **//** : cases when ant species were recorded on different branches of the same tree (two different territories on the same tree). For the inland transects we provide the trees’ code in the first column (see also Figure S1 for inland transect 1); for the coastal transect we provide only the number of trees.

| **Coastal transect** | **Tree family** | ***Tree species*** | ***Arboreal ant species recorded*** |
| --- | --- | --- | --- |
| 2 | Caesalpiniaceae | *Intsia bijuga* | *Technomyrmex* *albipes* |
| 3 | Clusiaceae | *Symphonia* sp.1 | *Technomyrmex* *albipes* |
| 1 |  | *Symphonia* sp.1 | *Technomyrmex* *albipes, Brachymyrmex* *cordemoyi* |
| 1 |  | *Symphonia* sp.1 | *Technomyrmex* *albipes*, *Camponotus* sp.4 |
| 5 | Combretaceae | *Combretum* sp.1 | *Technomyrmex* *albipes* |
| 1 |  | *Combretum* sp.1 | *Technomyrmex* *albipes*, *Brachymyrmex* *cordemoyi* |
| 1 |  | *Combretum* sp.1 | *Technomyrmex* *albipes*, *Camponotus* sp.4 |
| 50 | Lecythidaceae | *Barringtonia* *butonica* | *Technomyrmex* *albipes* |
| 5 |  | *Barringtonia* *butonica* | *Technomyrmex* *albipes*, *Brachymyrmex* *cordemoyi* |
| 3 |  | *Barringtonia* *butonica* | *Technomyrmex* *albipes*, *Brachymyrmex* *cordemoyi*, *Camponotus* sp.4 |
| 2 |  | *Barringtonia* *butonica* | *Technomyrmex* *albipes*, *Brachymyrmex* *cordemoyi,* *Tetraponera* *longula* |
| 5 |  | *Barringtonia* *butonica* | *Technomyrmex* *albipes*, *Camponotus* sp.4 |
| 2 (patches) | Malvaceae | *Hibiscus tiliaceus* | *Technomyrmex.* *albipes*, *Brachymyrmex* *cordemoyi*, *Camponotus* sp.4 |
| 1 (patches) |  | *Hibiscus tiliaceus* | *Technomyrmex* *albipes*, *Brachymyrmex* *cordemoyi,* *Tetraponera* *longula* |
| 1 |  | *Ceiba pentandra* | *Technomyrmex* *albipes*, *Brachymyrmex* *cordemoyi* |
| 1 |  | *Ceiba pentandra* | *Technomyrmex* *albipes*, *Brachymyrmex* *cordemoyi*, *Camponotus* sp.4 |
| 1 | Rhizophoraceae | *Anisophyllea fallax* | *Technomyrmex* *albipes* |
| 1 |  | *Anisophyllea fallax* | *Technomyrmex* *albipes*, *Brachymyrmex* *cordemoyi,* *Tetraponera* *longula* |
| 25 |  | *Bruguiera* *gymnorhiza* | *Technomyrmex* *albipes* |
| 3 |  | *Bruguiera* *gymnorhiza* | *Technomyrmex* *albipes*, *Brachymyrmex* *cordemoyi* |
| 2 |  | *Bruguiera* *gymnorhiza* | *Technomyrmex* *albipes*, *Brachymyrmex* *cordemoyi*, *Camponotus* sp.4 |
| 2 |  | *Bruguiera* *gymnorhiza* | *Technomyrmex* *albipes*, *Brachymyrmex* *cordemoyi*, *Tetraponera* *longula* |
| 1 |  | *Bruguiera* *gymnorhiza* | *Technomyrmex* *albipes*, *B.* *cordemoyi*, *Camponotus* sp.4, *Tetraponera* *longula* |
| 3 |  | *Bruguiera* *gymnorhiza* | *Technomyrmex* *albipes*, *Camponotus* sp.4 |
| 17 |  | *Bruguiera* *sexangula* | *Technomyrmex* *albipes* |
| 2 |  | *Bruguiera* *sexangula* | *Technomyrmex* *albipes*, *Brachymyrmex* *cordemoyi* |
| 1 |  | *Bruguiera* *sexangula* | *Technomyrmex* *albipes*, *Brachymyrmex* *cordemoyi*, *Camponotus* sp.4 |
| 1 |  | *Bruguiera* *sexangula* | *Technomyrmex* *albipes*, *Camponotus* sp.4, *Tetraponera* *longula* |
| 1 |  | *Macarisia pyramidata* | *Technomyrmex* *albipes* |
| 2 |  | *Macarisia pyramidata* | *Technomyrmex* *albipes*, *Camponotus* sp.4 |
| 1 | Euphorbiaceae | *Anthostema madagascariensis* | *Technomyrmex* *albipes*, *Brachymyrmex* *cordemoyi* |
| 1 |  | *Anthostema madagascariensis* | *Technomyrmex* *albipes*, *Brachymyrmex* *cordemoyi*, *Camponotus* sp.4 |
| 2 |  | *Uapaca* sp.1 | *Technomyrmex* *albipes* |
| **Epiphytes** | |  |  |
| on 48 trees | Melastomataceae | *Medinilla* sp. | *Technomyrmex* *albipes* |
| on 12 trees |  | *Medinilla* sp. | *Technomyrmex* *albipes*, *Brachymyrmex* *cordemoyi* |
| on 2 trees |  | *Medinilla* sp. | *Technomyrmex* *albipes*, *Brachymyrmex* *cordemoyi*, *Camponotus* sp.4 |
| on 2 trees |  | *Medinilla* sp. | *Technomyrmex* *albipes*, *Camponotus* sp.4 |
| * other ant species baited on *Medinilla* sp. | | | * *Leptothorax* sp.2 (3 trees); *Monomorium* sp.1 (3 trees); *Pheidole* sp.2 (3 trees); *Tapinoma subtile* (3 trees); *Tapinoma* sp.2 (3 trees); *Paratrechina* sp.1 (3 trees) |
| **Inland 1 transect** | **Tree family** | **Tree species** | **Arboreal ant species recorded** |
| A1 | Anacardiaceae | *Mangifera indica* | ( - ) |
| A96 |  | *Mangifera indica* | *Crematogaster madagascariensis* (dead, hollowed branches) |
| A68 |  | *Protorhus micronichia* | ( - ) |
| A87 |  | *Protorhus micronichia* | *Crematogaster kelleri*, *Monomorium* sp.1, *Camponotus* sp.3 |
| A52 |  | *Protorhus* sp.1 | ( - ) |
| A69 | Annonaceae | Unidentified (Annonaceae sp.1) | ( - ) |
| A67, A80 |  | *Xylopia burxifolia* | ( - ) |
| A55 |  | *Xylopia* sp.1 | ( - ) |
| A108, |  | *Xylopia* sp.1 | *Crematogaster kelleri* |
| A116, A119 |  | *Xylopia* sp.1 | *Crematogaster ranavalonae* (carton nest) |
| A5 c |  | *Landolfia* sp.1 (liana) | *Crematogaster* sp.1, *Monomorium* sp.1 |
| A5 | Burseraceae | *Cannarium madagascariensis* | *Crematogaster* sp.1, *Monomorium* sp.1 |
| A9, A74 |  | *Cannarium madagascariensis* | ( - ) |
| A42 |  | *Cannarium madagascariensis* | *Crematogaster madagascariensis*, *Crematogaster kelleri* |
| A59 |  | *Cannarium madagascariensis* | *Tapinoma subtile*, *Tetraponera* *longula* |
| A54 |  | *Cannarium* sp.1 | *Crematogaster kelleri*, *Camponotus* sp.1 (dead, hollowed branch) |
| A3 |  | *Protium madagascariensis* | ( - ) |
| A98 |  | *Protium* sp.1 | ( - ) |
| A76, A89 | Caesalpiniaceae | *Cynometra* sp.1 | ( - ) |
| A2 |  | *Intsia bijuga* | *Crematogaster rasoherinae*, *Crematogaster sewellii* (dead, hollowed branches) |
| A6 |  | *Intsia bijuga* | *Crematogaster* sp.1, *Leptothorax* sp.1 |
| A17, A25, A40 | A46 | *Intsia bijuga* | ( - ) |
| A37 |  | *Intsia bijuga* | *Crematogaster madagascariensis*, *Camponotus* sp.1 (dead, hollowed branches) |
| A104 | Clusiaceae | *Manea bongo* | *Crematogaster ranavalonae* |
| A14 |  | *Ochrocarpus* sp.1 | ( - ) |
| A19 |  | *Symphonia* sp.1 | *Brachymyrmex* *cordemoyi* |
| A28 |  | *Symphonia* sp.1 | ( - ) |
| A113 |  | *Symphonia* sp.2 | *Crematogaster ranavalonae* |
| A61 | Elaeocarpaceae | *Elaeocarpus* sp.1 | *Crematogaster rasoherinae*, *Crematogaster sewellii*, *Leptothorax* sp.3 |
| A50 |  | *Elaeocarpus* sp.1 | ( - ) |
| A84 | Euphorbiaceae | *Macaranga* sp.1 | *Camponotus* sp.1 (dead, hollowed branches) |
| A36 |  | *Uapaca louvelii* | *Crematogaster madagascariensis*, *Crematogaster kelleri* (dead, hollowed branches) |
| A63 |  | *Uapaca louvelii* | ( - ) |
| A65 |  | *Uapaca louvelii* | *Crematogaster kelleri*, *Tetraponera* *longula* |
| A94 |  | *Uapaca louvelii* | *Cr. madagascariensis*, *Cr. kelleri*, *Cr.* *ranavalonae* (carton nest), *Pheidole* sp.1 |
| A13 |  | *Uapaca* sp.1 | *Camponotus* sp.1 nr. *maculatus* (dead, hollowed branches) |
| A29 |  | *Uapaca* sp.1 | ( - ) |
| A30 |  | *Uapaca* sp.1 | *Crematogaster madagascariensis* (dead, hollowed branches) |
| A105 |  | *Uapaca* sp.1 | *Crematogaster ranavalonae*, Camponotus sp.2 |
| A109 |  | *Uapaca* sp.1 | *Crematogaster ranavalonae* (carton nest) |
| A103, A120 |  | *Uapaca* sp.2 | *Crematogaster ranavalonae* |
| A48 | Flacourtiaceae | *Homalium* sp.1 | ( - ) |
| A49 |  | *Homalium* sp.1 | ( - ) |
| A79, A85, |  | *Homalium* sp.1 | *Monomorium* sp.2 *termitobium complex* |
| A86 |  | *Homalium* sp.1 | *Crematogaster ranavalonae*, *Monomorium* sp.1 |
| A112 |  | *Homalium* sp.2 | *Crematogaster ranavalonae* |
| A111 |  | *Homalium* sp.3 | *Crematogaster ranavalonae* |
| A60 | Lauraceae | *Ocotea* sp.1 | *Crematogaster sewellii*, *Tetraponera* *longula* |
| A44, A45, A62 | A66 | *Ocotea* sp.1 | ( - ) |
| A64 |  | *Ocotea* sp.1 | *Crematogaster kelleri* |
| A70 |  | *Ocotea* sp.1 | *Crematogaster sewellii*, *Leptothorax* sp.3 |
| A72 |  | *Ocotea* sp.1 | *Crematogaster rasoherinae*, *Tapinoma subtile* |
| A73 |  | *Ocotea* sp.1 | *Crematogaster rasoherinae* |
| A75 |  | *Ocotea* sp.1 | *Leptothorax* sp.3 |
| A77 |  | *Ocotea* sp.1 | *Cr.* sp.1, *Cataulacus intrudens*, *Leptothorax* sp.1, *Leptoth.* sp.3, *Tetraponera* *longula* |
| A83 |  | *Ocotea* sp.1 | *Crematogaster kelleri* |
| A82 |  | *Ravensara* sp.1 | *Crematogaster kelleri* |
| A99 |  | *Ravensara* sp.1 | *Crematogaster ranavalonae* (carton nest) |
| A51 |  | Unidentified (Lauraceae sp.1) | ( - ) |
| A27 | Menispermaceae | *Brassaia madagascariensis* | *Crematogaster kelleri* |
| A78 | Mimosaceae | *Albizia* sp.1 | *Leptothorax* sp.3 |
| A81 |  | *Albizia* sp.1 | *Crematogaster kelleri* |
| A114 | Monimiaceae | *Tambourissia* sp. | *Crematogaster ranavalonae* |
| A32 | Moraceae | *Bosqueia* sp.1 | ( - ) |
| A90 |  | *Bosqueia* sp.1 | *Crematogaster madagascariensis*, *Pheidole* sp.1 |
| A93 |  | *Bosqueia* sp.1 | *Crematogaster madagascariensis*, *Crematogaster kelleri*, *Pheidole* sp.1 |
| A95 |  | *Bosqueia* sp.1 | *Crematogaster ranavalonae* (carton nest) |
| A106 |  | *Bosqueia* sp.1 | *Crematogaster ranavalonae* |
| A110 |  | *Bosqueia* sp.1 | *Crematogaster ranavalonae* |
| A101 |  | *Bosqueia* sp.2 | ( - ) |
| A33 |  | *Chlorophora* sp.1 | ( - ) |
| A8 |  | *Ficus* sp.1 | ( - ) |
| A97 |  | *Treculia* sp.1 | *Crematogaster rasoherinae*, *Cr. madagascariensis* (dead, hollowed branches) |
| A39 |  | Unidentified (Moraceae sp.1) | *Crematogaster kelleri* |
| A41 | Myristicaceae | *Bronchoneura* sp.1 | ( - ) |
| A10 |  | *Bronchoneura* sp.1 | *Crematogaster kelleri* |
| A35 | Myrtaceae | *Syzygium* sp.1 | *Crematogaster madagascariensis*, *Crematogaster kelleri* (dead, hollowed branches) |
| A38, A71 |  | *Syzygium* sp.1 | ( - ) |
| A91 |  | *Syzygium* sp.1 | *Crematogaster madagascariensis* |
| A107 |  | *Syzygium* sp.1 | *Crematogaster kelleri* |
| A115 |  | *Syzygium* sp.1 | *Crematogaster ranavalonae* (carton nest) |
| A23 | Papilionaceae | *Dalbergia madagascariensis* | ( - ) |
| A21 | Rhizophoraceae | *Anisophyllea fallax* | ( - ) |
| A118 |  | *Macarisia pyramidata* | *Crematogaster ranavalonae* (carton nest) |
| A17 b | Rubiaceae | *Bertiera* sp. (liana) | ( - ) |
| A100, A117 |  | Unidentified (Rubiaceae sp.1) | *Crematogaster ranavalonae* (carton nest) |
| A22, A31, A56 | Sapindaceae | *Allophyllum cobbe* | ( - ) |
| A11 |  | *Tinopsis* sp.1 | *Leptothorax* sp.1 |
| A18 | Sapotaceae | *Labramia louvelii* | *Crematogaster rasoherinae*, *Brachymyrmex* *cordemoyi* |
| A15, A43, A47 |  | *Labramia louvelii* | ( - ) |
| A16, A26 |  | *Mimusops* sp.1 | ( - ) |
| A4 | Sphaerosepalaceae | *Rhopalocarpus* sp.1 | *Crematogaster* sp.1, *Monomorium sp.1 termitobium* complex |
| A53 |  | *Rhopalocarpus* sp.1 | *Crematogaster kelleri*, *Tetraponera* *longula* |
| A58, A88 |  | *Rhopalocarpus* sp.1 | ( - ) |
| A7, A12 | Verbenaceae | *Vitex beraviensis* | *Crematogaster* sp.1 |
| A57 | Unidentified | Unidentified sp.1 | ( - ) |
| A20 |  | Dead, unidentified (1) | *Brachymyrmex* *cordemoyi* |
| A24 |  | Dead, unidentified (1) | *Camponotus* sp.1 (dead, hollowed branches) |
| A102 |  | Dead, unidentified (1) | *Crematogaster ranavalonae* |
| A92 |  | Dead, unidentified (1) | *Crematogaster madagascariensis*, *Tapinoma subtile*, *Camponotus* sp.1 |
| **Lianas** | |  |  |
| A5 b | Araceae | *Pothos scandens* (liana) | *Crematogaster* sp.1, *Monomorium* sp.1 |
| A1 b | Connaraceae | *Agelaea pentagyna* (liana) | ( - ) |
| A22 b | Dilleniaceae | *Dillenia tetracera* (liana) | ( - ) |
| A6 b | Piperaceae | *Peperomia* sp. (liana) | *Crematogaster* sp.1, *Leptothorax* sp.1 |
| **Inland 2 transect** | **Tree family** | **Tree species** | **Arboreal ant species recorded** |
| L4 | Anacardiaceae | *Campnosperma micrantea* | *Cr. madagascariensis*, *Cr. ranavalonae* **//** *Technomyrmex* *albipes* |
| L18 |  | *Campnosperma micrantea* | *Cr.* sp.3, *T*. *albipes*, *Leptoth.* sp.2, *B.* *cordemoyi*, *Paratrechina* sp.2, *Tapinoma subtile* |
| L68 |  | *Micronichia tsiramirang* | ( - ) |
| L34 |  | Unidentified (Anacardiaceae sp.1) | ( - ) |
| L50 |  | Unidentified (Anacardiaceae sp.1) | *Crematogaster madagascariensis*, *Technomyrmex* *albipes*, *Brachymyrmex* *cordemoyi* |
| L59 | Annonaceae | *Monantotaxis* sp.1 | ( - ) |
| L61 |  | *Xylopia buxifolia* | *Crematogaster ranavalonae* (carton nest) |
| L74 |  | *Xylopia* sp.1 | *Crematogaster madagascariensis* |
| L81 |  | *Xylopia* sp.1 | *Crematogaster ranavalonae* |
| L76, L77 | L78, L79 | *Xylopia* sp.1 | ( - ) |
| L20 | Apocynaceae | *Stephanostegia* sp.1 | ( - ) |
| L22 |  | *Stephanostegia* sp.1 | *Crematogaster madagascariensis* |
| L43 |  | *Stephanostegia* sp.1 | *Technomyrmex* *albipes*, *Brachymyrmex* *cordemoyi* |
| L56 |  | *Stephanostegia* sp.1 | *Crematogaster ranavalonae*, *Brachymyrmex* *cordemoyi*, *Paratrechina* sp.2 |
| L85 | Burseraceae | *Cannarium madagascariensis* | *Crematogaster ranavalonae* (carton nest) |
| L63 | Caesalpiniaceae | *Dialium madagascariensis* | *Crematogaster ranavalonae* |
| L8 | Clusiaceae | *Calophyllum* sp.1 | *Paratrechina* sp.1, *Paratrechina* sp.2 |
| L12, L27 | L19 | *Calophyllum* sp.1 | ( - ) |
| L36 |  | *Calophyllum* sp.2 | *Brachymyrmex* *cordemoyi* |
| L45 |  | *Calophyllum* sp.1 | *Technomyrmex* *albipes*, *Paratrechina* sp.1, *Paratrechina* sp.2, *Tetramorium bicarinatum* |
| L53 |  | *Calophyllum* sp.2 | *Cr. madagascariensis*, *Monomorium* sp.2, *Paratrechina* sp.2, *B.* *cordemoyi*, *T.* *albipes* |
| L70, L86 |  | *Calophyllum* sp.2 | *Crematogaster ranavalonae* |
| L67 |  | *Calophyllum* sp.2 | ( - ) |
| L6 |  | *Rheedia* sp.1 | *Crematogaster ranavalonae* **//** *Technomyrmex* *albipes*, *Paratrechina* sp.2 |
| L21 |  | *Rheedia* sp.1 | *Technomyrmex* *albipes*, *Brachymyrmex* *cordemoyi* |
| L24 |  | *Rheedia* sp.1 | *Tapinoma subtile* |
| L87 |  | *Rheedia* sp.1 | *Crematogaster ranavalonae* |
| L7 |  | *Symphonia* sp.1 | *Cr. rasoherinae* **//** *T. Albipes*, *Monomorium* sp.3, *Paratrechina* sp.1, *Parat.* sp.2 |
| L10 |  | *Symphonia* sp.1 | *Crematogaster madagascariensis*, *Technomyrmex* *albipes* |
| L38 |  | *Symphonia* sp.1 | *Cr. madagascariensis*, *Leptothorax* sp.4, *Brachymyrmex* *cordemoyi*, *Paratrechina* sp.1 |
| L55 |  | *Symphonia* sp.1 | *Paratrechina* sp.2, *Tetramorium lanuginosum*, *Brachymyrmex* *cordemoyi* |
| L75 |  | *Symphonia* sp.1 | ( - ) |
| L80, L88 |  | *Symphonia* sp.1 | *Crematogaster ranavalonae* |
| L84 |  | *Symphonia* sp.1 | *Crematogaster ranavalonae* **//** *Technomyrmex* *albipes* |
| L13 |  | *Symphonia* sp.2 | *Technomyrmex* *albipes* |
| L26 |  | *Symphonia* sp.2 | *Crematogaster kelleri*, *Monomorium* sp.1, *Brachymyrmex* *cordemoyi* |
| L89 |  | *Symphonia* sp.2 | ( - ) |
| L30 |  | *Symphonia* sp.3 | ( - ) |
| L32 |  | *Symphonia* sp.3 | *Technomyrmex* *albipes*, *Brachymyrmex* *cordemoyi*, *Paratrechina* sp.1 |
| L35, L58 | Combretaceae | *Combretum* sp.1 | ( - ) |
| L17 | Ebenaceae | *Diospyros* sp.1 | *Crematogaster ranavalonae*, *Leptothorax* sp. 2 |
| L16 | Eleocapaceae | *Sloanea rhodonta* | *Technomyrmex* *albipes* |
| L42 | Erythroxylaceae | Unidentified (Erythroxylac. sp.1) | *Cr. ranavalonae* (carton nest), *B.* cordemoyi, *Paratrechina* sp.2, *Tetramorium bicarinatum* |
| L52 |  | Unidentified (Erythroxylac. sp.1) | *Crematogaster madagascariensis*, *Paratrechina* sp.2 |
| L54 |  | Unidentified (Erythroxylac. sp.1) | *Paratrechina* sp.2, *Tetramorium lanuginosum* |
| L2 | Euphorbiaceae | *Anthostema madagascariensis* | *Crematogaster ranavalonae* (carton nest) |
| L14 |  | *Anthostema madagascariensis* | *Crematogaster ranavalonae* (carton nest) |
| L25 |  | *Anthostema madagascariensis* | *Leptothorax* sp.2, *Solenopsis* *mameti* |
| L28 |  | *Anthostema madagascariensis* | *Cr. kelleri*, *Cr. ranavalonae* **//** *Leptothorax* sp.2, *B.* *cordemoyi*, *T.* *albipes* |
| L29 |  | *Anthostema madagascariensis* | *Tapinoma subtile* |
| L37 |  | *Anthostema madagascariensis* | *Brachymyrmex* *cordemoyi*, *Paratrechina* sp.2 |
| L40 |  | *Anthostema madagascariensis* | *Pheidole* sp.1, *Tetramorium bicarinatum*, *Paratrechina* sp.1, *Paratrechina* sp.2 |
| L44 |  | *Anthostema madagascariensis* | *Technomyrmex* *albipes*, *Brachymyrmex* *cordemoyi*, *Tetramorium bicarinatum* |
| L46 |  | *Anthostema madagascariensis* | *Brachymyrmex* *cordemoyi*, *Paratrechina* sp.1 |
| L47 |  | *Anthostema madagascariensis* | *Technomyrmex* *albipes*, *Brachymyrmex* *cordemoyi*, *Paratrechina* sp.1, *Tapinoma subtile* |
| L48 |  | *Anthostema madagascariensis* | *Technomyrmex* *albipes*, *Brachymyrmex* *cordemoyi*, *Tapinoma subtile* |
| L71 |  | *Anthostema madagascariensis* | *Crematogaster kelleri* |
| L15 |  | *Macaranga* sp.1 | *Crematogaster madagascariensis*, *Brachymyrmex* *cordemoyi* |
| L9, L33 |  | *Uapaca* sp.1 | *Technomyrmex* *albipes, Brachymyrmex* *cordemoyi* |
| L31 |  | *Uapaca* sp.1 | *Paratrechina* sp.1, *Technomyrmex* *albipes* |
| L39 |  | *Uapaca* sp.1 | *Crematogaster madagascariensis*, *Tapinoma subtile* |
| L49 |  | *Uapaca* sp.1 | *Tapinoma subtile* |
| L73 |  | *Uapaca* sp.1 | ( - ) |
| L82, L83 |  | *Uapaca* sp.1 | *Crematogaster ranavalonae* (carton nest) |
| L69 |  | *Uapaca* sp.2 | ( - ) |
| L72 |  | *Uapaca* sp.2 | *Crematogaster ranavalonae* (carton nest) |
| L3 | Lauraceae | *Ocotea* sp.1 | *Crematogaster madagascariensis*, *Crematogaster ranavalonae* |
| L41 |  | *Ravensara* sp.1 | *Paratrechina* sp.1 |
| L57 |  | *Ravensara* sp.1 | *Cr. ranavalonae*, *Brachymyrmex* *cordemoyi*, *Paratrechina* sp.2, *Tetramorium bicarinatum* |
| L65 | Lecythidaceae | *Fetida* sp.1 | *Crematogaster ranavalonae* |
| L11 | Papilionaceae | *Cordyla haraka* | *Cr. madagascariensis*, *Cr. ranavalonae*, *Monomorium* sp.3 **//** *Technomyrmex* *albipes* |
| L66 |  | *Cordyla haraka* | *Crematogaster ranavalonae* |
| L23 |  | *Dalbergia maritime* | ( - ) |
| L64 | Rubiaceae | *Fernelia* sp.1 | *Crematogaster madagascariensis*, *Crematogaster ranavalonae* |
| L5 | Sapotaceae | *Capurodendron* sp.1 | *Cr. ranavalonae* **//** *T.* *albipes*, *Pheidole* sp.1, *Paratrechina*.sp.1, *Tapinoma subtile* |
| L51 |  | *Faucherea sambiranniensis* | *Crematogaster madagascariensis*, *Brachymyrmex* *cordemoyi*, *Technomyrmex* *albipes* |
| L60 |  | *Faucherea parviflora* | *Crematogaster ranavalonae* (carton nest) |
| L62 |  | *Mimusops perrieri* | *Crematogaster ranavalonae* (carton nest) |
| L1 |  | *Syderoxylon* sp.1 | ( - ) |
| **Epiphytes and lianas** | |  |  |
| L60 b | Melatomataceae | *Medinilla* sp.1 (epiphyte) | *Pheidole* sp.1 |
| L27 b | Apocynaceae | *Landolfia* sp.1 (liana) | ( - ) |
| L21 b | Asclepiadaceae | *Secamon* sp.1 (liana) | *Technomyrmex* *albipes*, *Brachymyrmex* *cordemoyi* |
| L59 b | Connaraceae | *Agelaea* sp.1 (liana) | ( - ) |

(patches) *Hibiscus tiliaceus* develops in patches of 6 to 10 m along the shore.
