# Supplementary material for: Spatial Distribution of Dominant Arboreal Ants in a Malagasy Coastal Rainforest: Gaps and Presence of an Invasive Species
Source: PLoS One. 2010 Feb 19;5(2):e9319. doi: 10.1371/journal.pone.0009319 (PMC2824834; doi:10.1371/journal.pone.0009319)
Supplement: Supplementary file 2 [file pone.0009319.s001.doc]

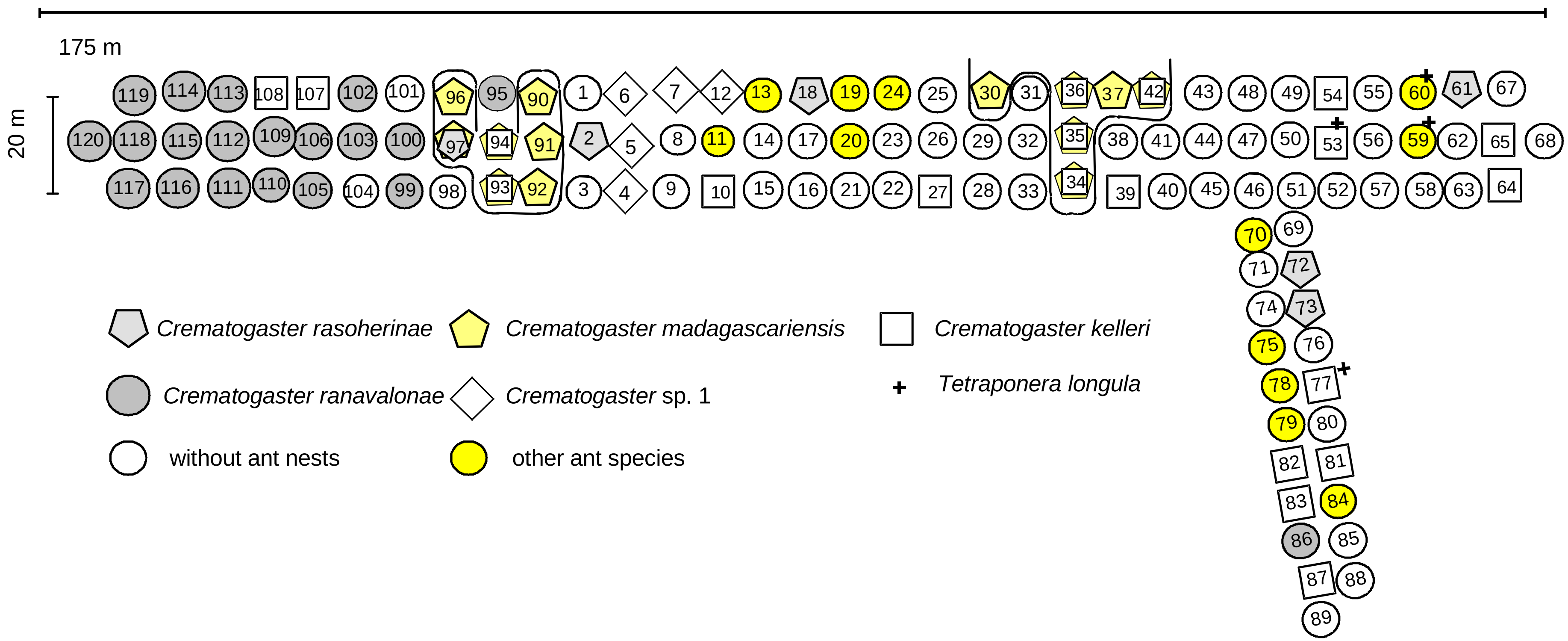
175 m
20 m
Crematogaster rasoherinae
Crematogaster madagascariensis
Crematogaster kelleri
Crematogaster ranavalonae
Crematogaster sp. 1
Tetraponera longula
without ant nests
other ant species
